# Urinary Potential Renal Acid Load (uPRAL) among Vegans Versus Omnivores and Its Association with Bone Health in the Cross-Sectional Risks and Benefits of a Vegan Diet Study

**DOI:** 10.3390/nu14214468

**Published:** 2022-10-24

**Authors:** Katharina J. Penczynski, Thomas Remer, Juliane Menzel, Klaus Abraham, Cornelia Weikert

**Affiliations:** 1 Department of Food Safety, German Federal Institute for Risk Assessment, 10589 Berlin, Germany; 2 DONALD Study Centre Dortmund, Institute of Nutrition and Food Science (IEL), University of Bonn, 44225 Dortmund, Germany; 3 Institute of Social Medicine, Epidemiology and Health Economics, Charité–Universitätsmedizin Berlin, Corporate Member of Freie Universität Berlin and Humboldt-Universität zu Berlin, 10117 Berlin, Germany

**Keywords:** 24 h urine, BUA, cross-sectional study, QUS, urinary phosphate, urinary PRAL, urinary sulfate, vegan

## Abstract

Both veganism and high dietary acid load are linked to unfavorable bone health. However, the specific role of dietary alkali or acid load for the bone health of vegans is so far unknown. Thus, the renal biomarker for dietary acid or alkali load, i.e., urinary potential renal acid load (uPRAL), was measured in 24 h urine samples of 34 vegans and 35 omnivores (50.7% males). Bone health was assessed via calcaneal quantitative ultrasound. Associations between uPRAL and bone health indices were examined using multivariable general linear models. Compared to omnivores, vegans had a significantly lower uPRAL (mean difference = −34.5 mEq/24 h, *p <* 0.0001), a lower 24 h urinary phosphate excretion (*p =* 0.0004), a lower 24 h urinary sulfate excretion (*p =* 0.01), and a higher urine pH value (*p <* 0.0001). Broadband ultrasound attenuation (BUA) was lower among vegans versus omnivores (*p =* 0.037), yet it was not associated with uPRAL irrespective of adjustments. This study confirms different acid-base profiles of vegans and omnivores, with a pronounced alkaline excess among vegans and a rather low acid load among a group of omnivores with moderate protein intake. Within this spectrum of alkaline to low acid load, no association with bone health was found.

## 1. Introduction

Vegan diets, which exclude any food of animal sources, have increased in popularity, but they may entail diverse health implications. Amongst the most prominent health concerns associated with veganism is lower bone health [1], as indicated by lower bone mineral density (BMD) and higher fracture rates among vegans compared to omnivores in two meta-analyses of observational studies [2,3].

Compared to typical Western omnivorous diets, vegan diets are usually characterized by lower protein and higher fruit and vegetable intakes—diet components with established importance for the acid-base status [4]. Accordingly, studies estimating acid load from dietary intake data report a lower acid load of vegan diets [5,6,7,8].

Yet, multiple approaches exist for the assessment of acid load, which can be based on either dietary intake or urinary excretion. A key advantage of urinary excretion-based approaches—such as urinary potential renal acid load (uPRAL)—over dietary-based approaches is their ability to reflect actual intakes and the individual bioavailability of acid-base relevant anions and cations [9]. Hence, uPRAL is considered a direct biomarker of diet-dependent acid load [9]. To our knowledge, this valuable approach has not been used so far to investigate the presumed difference in diet-dependent acid load between vegans and omnivores.

Dietary-induced shifts in acid-base balance with mild systemic pH and bicarbonate adaptations still within the physiological range are thought to induce small endocrine-metabolic changes with diverse long-term health consequences, including bone health [9,10]. Although it remains a topic of debate kindled by conflicting results [11], notable evidence links high dietary acid load to unfavorable bone parameters or fractures [12,13,14,15]. Interestingly, it was argued that the low acid load of vegan diets would protect vegans’ bones [4], at least in a state of sufficient nutrient supply [16]. Yet on the contrary, it has also been reasoned that benefits of alkalization for bone status may be confined to people consuming diets with pronounced acid load and/or people with relevant predispositions, e.g., kidney dysfunction, metabolic syndrome, frailty or osteopenia [11,17,18]. The latter is an interesting argumentative approach, since it would imply that vegans, with their presumably already low dietary acid load, may not additionally profit from further dietary alkalization with regard to bone health. Hence, the complex association of vegan diet, dietary acid or alkali load and bone health needs to be further investigated.

Against this background, the present study aimed to:

(i) Characterize uPRAL, 24 h urinary excretion profiles of acid-base relevant ions and urinary pH of vegans in comparison to omnivores.
(ii) Investigate if the expected very low acid, i.e., alkaline, loads of vegan diets may contribute to alleviating the unfavorable association of veganism with poor bone health.

## 2. Materials and Methods

### 2.1. Design and Study Population

The “Risks and Benefits of a Vegan Diet” (RBVD) study is a cross-sectional study conducted at the study center of the German Federal Institute of Risk Assessment (BfR) in Berlin, Germany, from January to July 2017. The study was approved by the Ethics Committee of the Charité-Universitätsmedizin Berlin (No. EA4/121/16) and written informed consent was obtained from all participants.

Details on the RBVD study were published previously [19]. Briefly, recruitment of participants was based on announcements in selected grocery stores and restaurants in Berlin (Germany), followed by a screening for eligibility criteria, and a sex- and age-matched selection of 36 vegans and 36 omnivores (for sample size calculations see [20]). Eligibility criteria included: age of 30–60 years, strictly vegan (excluding any animal-based foods) or omnivorous diet (minimal weekly consumption of three servings of meat or two servings of meat plus two servings of processed meat products), minimal diet adherence of 1 year, BMI < 30 kg/m^2^, absence of current infection or serious diseases (such as cancer, myocardial infarction, stroke, diabetes), absence of medication with glucocorticoids or proton pump inhibitors, absence of pregnancy or breastfeeding. The primary outcome of the RBVD study was the difference in the bone health parameter broadband ultrasound attenuation (BUA) between vegans and omnivores. The samples size was calculated to achieve a power of 80% at a significance level of α = 0.05 to discern a clinically relevant difference in BUA of at least 5% [19,20]. Acid-base parameters represent secondary outcomes.

Due to missing data in 24 h urinary excretions of sodium and/or potassium, three participants were excluded, resulting in a data set including 69 participants for uPRAL analyses. The data set for bone health analyses included *n* = 68 due to the exclusion of one further participant, as explained below.

### 2.2. Bone Strength and Microstructure Parameters

Quantitative ultrasound (QUS) parameters of bone strength and microstructure (BUA [dB/MHz], speed of sound (SOS [m/s]) and stiffness index (SI)) were estimated twice bilaterally at the *os calcaneus* by trained study staff using Achilles EXPII (General Electric Healthcare, Little Chalfont, UK). Anatomical or medical conditions (ankle edema, trauma, or fracture) hampering a valid QUS measurement resulted in unilateral measures in the case of *n* = 4 participants and complete exclusion of *n* = 1 participant. For statistical analysis, arithmetic means were calculated of all available measurements.

### 2.3. Urine Sampling and Analysis

Participants were instructed on the standard procedures of 24 h urine collection. The 24 h urine sample had to be collected in provided preservative-free plastic containers starting on the day preceding the study visit (excluding the first morning micturition) and ending on the morning of the study visit (including the first morning micturition). At the study center, collected samples were mixed, aliquoted, and analyzed on the same day or stored at −80 °C for subsequent analyses. Same-day analysis included measurement of urine pH at the study center using a pH meter (Knick Portamess, Berlin, Germany) in addition to external measurements of urinary concentrations of calcium and magnesium by flame atomic absorption spectrometry (AAS NovAA ^®^350), as well as sodium and potassium by ion-selective electrodes at the Labor28 GmbH (Berlin, Germany). Subsequent analysis in 2021 included measurement of urine concentrations of sulfate, phosphate and chloride by Dionex 2000i/SP ion chromatography with an ion Pac AS4A column (Dionex GmbH, Idstein, Germany) at the laboratory of the DONALD Study (Dortmund, Germany).

uPRAL was calculated from 24 h urinary ion excretions according to the following equation of Remer and Manz [21]:

(1) $$\begin{array}{l} \mathrm{uPRAL}\;\left(\mathrm{mEq}/24\;h\right)\\ \;\;\;\;\;\;\;=\mathrm{Cl}\;\left(\mathrm{mmol}/24\;h\right)+2{*\mathrm{SO}}_{4}\left(\mathrm{mmol}/24\;h\right)+1.8{*\mathrm{PO}}_{4}\left(\mathrm{mmol}/24\;h\right)\\ \;\;\;\;\;\;\;-\mathrm{Na}\;\left(\mathrm{mmol}/24\;h\right)-K\;\left(\mathrm{mmol}/24\;h\right)-2*\mathrm{Mg}\;\left(\mathrm{mmol}/24\;h\right)-2\\ \;\;\;\;\;\;\;*\mathrm{Ca}\;\left(\mathrm{mmol}/24\;h\right) \end{array}$$

### 2.4. Dietary Intake Assessment

Dietary intake was assessed by three-day weighed dietary records, capturing two weekdays and one weekend day. Participants were instructed to weigh every consumed item as well as leftovers with a provided electronic food scale and to record detailed information on the item and its preparation. Daily nutrient intakes, i.e., total energy, protein, and alcohol, were calculated using the EAT-Software (University of Paderborn, 3.5.5), which is based on data from the German Nutrient Database (Bundeslebensmittelschlüssel (BLS), version 3.02) and represents averages over the three recorded days.

Identification of potential misreporting was based on the relation of recorded total energy intake to estimated basal metabolic rate [22], applying established cut-offs for underreporting [23] and overreporting [24].

### 2.5. Assessment of Covariates

Anthropometric measurements were taken in duplicate by trained staff according to standard procedures, with participants being barefoot and in underwear. Standing height (cm) was determined using a flexible stadiometer (SECA, Hamburg, Germany) and body weight (kg) was measured using an electronic digital scale (Omron BF511, Omron Healthcare Ltd., Kyoto, Japan). Waist circumference was measured at the midpoint between lower ribs and iliac crest. Duplicate measurements were averaged and used for calculation of body mass index (BMI, kg/m^2^).

Questionnaires were used for inquiry of educational attainment, smoking habits, current and previous diseases, family disease history, intake of medication or supplements, and physical activity. Physical activity was created and categorized according to the “recreational index” of the InterAct Consortium [25], which is based on sum duration of walking, cycling and sports (averaged for summer and winter, in h/week) multiplied by standard metabolic equivalent of task (MET) estimates (3.0 for walking and 6.0 for cycling and sports). Categories were then dichotomized to represent moderate to high physical activity (yes/no) corresponding with >33.75 MET h/week.

### 2.6. Statistical Analyses

Statistical analyses were conducted with the SAS statistical software package version 9.4 (SAS Institute Inc., Cary, NC, USA).

Characteristics of the study population are presented as mean ± SD or median (25th, 75th percentile) for normal or non-normal continuous variables, respectively, and as absolute frequencies (percentages) for categorical variables. Tests for differences between vegans and omnivores included independent two-sample Student’s *t* test or Satterthwaite *t* test (in case of heteroscedasticity) for normal continuous variables, and Mann–Whitney U test for non-normal continuous variables. Associations of uPRAL with urine pH and of dietary protein intake with 24 h urinary excretions of phosphate and sulfate were analyzed by Spearman correlations.

Multivariable linear associations between uPRAL and bone health outcomes (primary outcome: BUA) were analyzed in general linear models (PROC GLM) and compared to robust regressions (PROC ROBUSTREG).

Basic models (model A) represent unadjusted models including the predictor uPRAL only. Adjusted models (models B to D) were constructed by inclusion of a priori fixed covariates along with hierarchical inclusion of covariates, significantly predicting the outcome and substantially modifying the association (change of *β*_uPRAL_ ≥ 10%). A priori covariates included sex, age, smoking status (current, ex, non-smoker), BMI (kg/m^2^), moderate to high physical activity (yes/no), alcohol intake (g/d), protein intake (g/d), and 24 h urinary calcium excretion (mmol/24 h). Covariates considered in the hierarchical approach comprised veganism (yes/no), high educational attainment (yes/no), season of study visit (January to March/April to July), total energy intake (MJ/d), duration of veganism (months), urine volume (L/24 h), use of oral contraceptives, antirheumatic or antihypertensive medication (yes/no), and supplementation with calcium, magnesium or vitamin D (yes/no). For comparability, identical adjusted models were used for all outcomes. Results of multivariable linear associations are presented as adjusted least-square means (95%-CI) of bone health outcomes in sex-specific tertiles of uPRAL along with parameters of linear trends (*p*_trend_, *β*_trend_, *SE*_trend_) from models with uPRAL as a continuous variable.

Sensitivity analyses separately investigated the relevance of menopausal status, under- and overreporting (yes/no), and prevalence of rheumatic or thyroid diseases (yes/no) by additional inclusion of the respective variable to the fully adjusted models.

## 3. Results

Characteristics of the study sample (*n* = 69) are presented in Table 1. Among the 34 vegans included in this analysis, veganism was followed for 4.9 years in median. No significant differences in sex, age, or socioeconomic and lifestyle factors were detected between both diet groups, except for lower BMI among vegans as compared to omnivores. Most importantly, vegans were characterized by significantly lower uPRAL and higher urine pH (both *p <* 0.0001).

**Figure 1:**
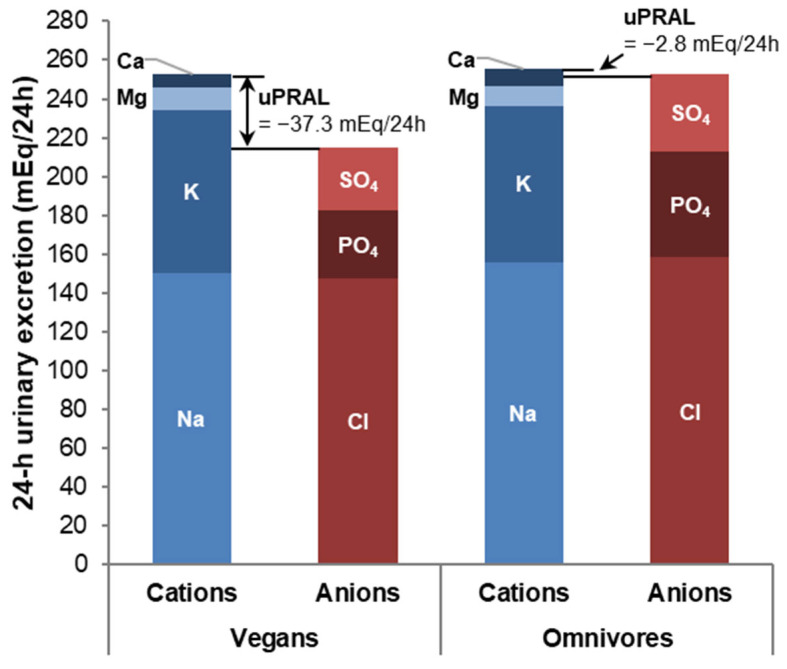
Urine ionogram of mean 24 h urinary excretions of cations and anions related to uPRAL (in mEq/24 h) among 34 vegans and 35 omnivores.

As is noticeable in the urine ionogram (Figure 1), the lower uPRAL of vegans was mainly driven by lower phosphate and sulfate excretions (*p =* 0.0004 and *p =* 0.01, respectively), which corresponded with lower protein intake among vegans vs. omnivores (*p =* 0.02; Table 1; correlation with protein intake: *r*_Spearman_ = 0.41, *p =* 0.0005 for phosphate and *r*_Spearman_ = 0.63, *p <* 0.0001 for sulfate).

**Figure 2:**
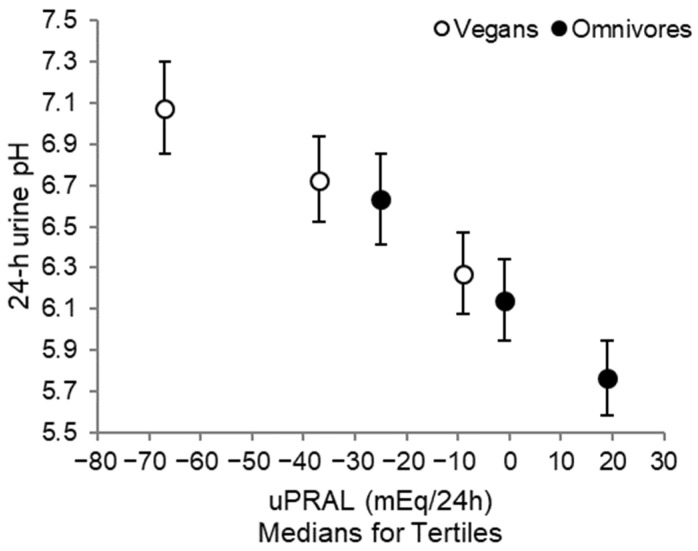
Geometric means and 95%-CI of 24 h urine pH in tertiles of uPRAL for vegans (*n* = 34) and omnivores (*n* = 35).

Further, a strong inverse correlation between uPRAL and urine pH was evident (*r*_Spearman_ = −0.85, *p <* 0.0001; Figure 2).

Among the parameters of bone health, BUA was significantly lower among vegans in comparison to omnivores (*p =* 0.037; *n* = 68; Table 1), which was independent of sex, age, and BMI (*p =* 0.045).

No significant association was observed between uPRAL and any QUS parameter (BUA, SOS or SI) irrespective of adjustment for veganism, sex, age, BMI, smoking status, physical activity, urinary calcium excretion, alcohol consumption, and protein intake (Table 2). Robust regression and sensitivity analyses (considering under- or overreporting, rheumatic or thyroid diseases, and menopausal status) produced grossly similar results.

Of note, as shown in Table 2, five vegans fall into the third uPRAL tertile, equivalent to moderate acid load (median uPRAL = 14.1 mEq/24 h), and three omnivores fall into the first uPRAL tertile, equivalent to pronounced alkali load (median uPRAL = −53.6 mEq/24 h).

## 4. Discussion

The present study has characterized uPRAL, 24 h urinary ion excretions and urine pH among vegans and omnivores and investigated the specific role of dietary acid or alkali load for bone health parameters in the healthy participants of the RBVD study. Our findings demonstrate and confirm that pronounced alkali load and higher urine pH result from vegan versus omnivorous diets. Further, the present biomarker-based results indicate that higher alkali loads among vegans can be attributable, to a relevant degree, to a lower phosphate and sulfur intake corresponding with lower protein intakes. Despite the clear differences in uPRAL and urinary pH between both diet groups, the omnivores of our study were also characterized by a rather low dietary acid load. Thus, as an important finding, no association between dietary alkalinity and bone health parameters could be detected for this particular range from low acid to high alkaline load.

### 4.1. Acid Load of Vegan Diets

In comparison to vegetarian or omnivorous diets, vegan diets are reported to comprise the lowest acid loads, as estimated by algorithms based on dietary intake data [5,6,7,8,26]. Yet, this is the first study to corroborate a pronounced difference in uPRAL among vegans versus omnivores based on urinary excretion data.

Previous studies have reported a higher mean urine pH among vegans (6.2–6.7) as compared to omnivores (5.7–6.2), corresponding to the lower acid load of their diets [5,8], which is also in line with our results. Yet, to our knowledge, no other study investigated urinary excretion profiles of ions relevant to acid load among vegans. The observed lower 24 h urinary excretion of phosphate and sulfate corresponds well with the lower protein intake among vegans of our study.

The few vegans in the third uPRAL tertile and few omnivores in the first uPRAL tertile demonstrate that vegan diets are not inevitably alkalizing and omnivorous diets are not inevitably acidifying, highlighting the importance of food choices with regard to a balanced and varied diet.

### 4.2. Bone Health

Although a favorable low acid load of vegetarian and vegan diets has been presumed to counteract their bone-detrimental effect [4], this hypothesis, especially among vegans, has not been tested to date.

In general agreement with our results, lower bone health (lower BMD levels and higher fracture rates) among vegans over omnivores was reported in a recent review [1], two meta-analyses of observational studies [2,3] and a recent prospective study [27]. A biomarker pattern of multiple bone-relevant nutrients and hormones appeared important to bone health in our previous analysis of the RBVD data [20]. Among the major contributors to the pattern were urinary calcium and magnesium, two principal constituents of the acid-base system.

Bone loss in metabolic acidosis is a long-known phenomenon [28,29] attributable to a direct effect of extracellular protons on osteoclasts, osteoblasts and matrix proteins and an indirect effect via deregulation of the growth hormone/IGF-axis and elevated glucocorticoids [9,10,30]. Similar, yet more subtle, cellular and endocrine-metabolic effects on bones have been observed with dietary-induced low-grade acidotic states well within the physiological range [9,10]. These rather small effects are expected to culminate in impaired bone health if net acid-producing diets are maintained habitually for an extended time [9,10,11].

Despite the biological plausibility for the bone-detrimental effect of dietary acid load and several findings of links to fracture risk or prediction scores [13,14,15], evidence appears inconsistent [11]. Upon closer consideration of our results, however, it is highly relevant that our study population was not only healthy but also captured a rather low spectrum of dietary acid load, reaching from highly negative (i.e., alkaline) to only moderately positive (i.e., acidic) uPRAL values. Our null findings in this low spectrum of uPRAL might be interpreted as indirect support for the findings indicating that bones benefit from alkalization in predisposed individuals, e.g., with renal functional losses or subclinical acidosis (mild shifts of blood pH and bicarbonate towards the lower range), or in subjects habitually consuming high-PRAL diets [11,17,18]. Further, the rather low protein intake of our study population merits attention. Especially the combination of low protein intake and low dietary acid load, as in our study, appeared to decouple potential acid load effects from bone health [18,31]. Notwithstanding, it needs to be stressed that our study was not designed to test any of those hypotheses and therefore these possible explanations remain speculative.

Among other reasons discussed for conflicting findings [9,11,32], methodological differences appear most convincing. The detection of the rather small effects of low-grade metabolic acidosis on bone health demands detailed high-quality data and a thorough understanding of all relevant factors influencing the association between acid load and bone health. To begin with, only sufficiently detailed assessment of dietary acid load—ideally based upon repeated 24 h urinary excretion of acid-base biomarkers—will ensure an appropriate reflection of habitual low-grade acidosis [9]. Direct measurement of net acid excretion (NAE, via 24 h urinary ammonium, titratable acids and bicarbonate excretions) is generally considered as the best available measure of net endogenous acid production [9]. Yet, use of 24 h urinary ion excretions for the calculation of uPRAL in our study represents a good and well-established biomarker, which specifically captures the dietary impact on the acid-base status [9]. Using uPRAL instead of NAE, however, omits dietary noncombustible organic acids (e.g., phenolic, quinic, or benzoic acids), which will conceivably differ between diet groups consuming different amounts of fruit and vegetables. Since fruit and vegetables are not only sources of organic acids but also main contributors to alkalization, the net contribution of organic acids is not of primary relevance [31]. A specific limitation considering our acid-base assessment is the use of a single 24 h urine sample, which can only capture a short snapshot of exposure.

Moreover, in order to discern the specific impact of dietary acid load on bone health, it is of the utmost importance to consider the complex interrelations of all influential factors. Adjustment for either protein intake or its biomarker, urinary nitrogen excretion, appears particularly mandatory. Protein is known to elicit counter-directed effects on bone: a bone-detrimental effect through contribution to acid load on the one side and a strong direct bone-anabolic effect on the other side. A lack of consideration of this complex bi-directional role of proteins for bone metabolism may lead to partial or complete masking of associations [9,33,34].

Finally, different methods for assessment of bone health (peripheral quantitative computed tomography (pQCT), dual-energy X-ray absorptiometry (DEXA), and QUS) all associate—although to different degrees—with osteoporosis and fracture risk [35,36,37,38,39]. Assessment of bone health by QUS in our study instead of the elaborate pQCT or the widely applied DEXA may be seen as a limitation of our study, yet these methods partly reflect different properties of bone (areal vs. volumetric BMD, bone mass, size, structure, elasticity and compartment-specific properties) [35,38,39]. Up to now, it is not fully elucidated if and to what extent the effects of low-grade metabolic acidosis are specific to bone site, compartment (trabecular or cortical), mass and structural properties of bone or differ in specific age spans and across sexes. Generally, endocrinological and nutritional influences are assumed to act site-unspecifically [38]. However, differential changes in cortical and trabecular compartments were observed in ammonium chloride-induced chronic metabolic acidosis among rats [40]. Compartment-specific bone loss was further reported in the growing and mature skeleton (following immobilization) [38] and throughout aging [41]. Whereas the growing skeleton adapts to disuse at the periosteal surface, the mature skeleton reacts on endocortical and trabecular surfaces [38]. Throughout aging, trabecular bone is continuously lost, starting in young adulthood, possibly following a declining course of the IGF-system, while cortical bone is lost mainly upon menopause in women and in elderly men, resulting from the evolving sex-steroids deficiency [41]. Congruent with this compartment-specificity across age, prospective studies in youths found associations of dietary acid load with cortical area (apart from BMC) [13,31,33], whereas two [12,15] out of three compartment-specific studies among adults [12,15,42] indicated predominant effects on trabecular bone. Three studies performing calcaneal QUS-measurements reported inverse associations between dietary PRAL and BUA among women (partly restricted to those with a fracture history) [14,43,44]. Given that 95% of the calcaneus is composed of trabecular bone [35,37], calcaneal BUA also mainly reflects trabecular properties. Of note, the effect sizes in these studies were relatively low (1.5–3% lower BUA for highest versus lowest quantiles of acid load) [14,43,44]. Thus, our non-significant inverse association might also be explained, at least in part, by insufficient power to detect such a small difference (a sample size of 72 was calculated as necessary to discern a 5% difference in BUA between vegans and omnivores [20]). Apart from the specific limitations extensively discussed above, the relatively small and non-representative convenience sample and the cross-sectional study design also call for consideration.

The major strengths of our study include the successful matching of recruited vegans and omnivores, accurate and standardized data assessment, especially biomarker measurement of dietary acid or alkali load based on 24 h urinary anion and cation excretions, and the investigation of its association with bone health parameters.

## 5. Conclusions

Our study provides an elaborate characterization of uPRAL, 24 h urinary ion excretions and urinary pH among healthy vegans and omnivores. It confirms different acid-base profiles of both diet groups, with a pronounced alkaline excess among vegans and a balanced to low acid load among the omnivores of our study. Further, it demonstrates that within this spectrum of alkaline to low acid load, combined with low to moderate protein intakes, no benefits for bone health could be seen.

It is important to note that a vegan diet is not per se alkalizing. With unfavorable food choices, the benefits of the alkali load of usual vegan diets can be lost.

## Figures and Tables

**Table 1:** Characteristics of vegans and omnivores (*n* = 69).

Characteristics	*n*	Vegans	Omnivores	*p*
* n*	69	34	35	
Sex (*n*, % females)	69	17 (50%)	17 (48.6%)	0.9
Age (years)	69	36.5 (32.0, 41.0)	38.0 (32.0, 46.0)	0.7
Anthropometric data				
BMI (kg/m^2^)	69	22.2 (20.3, 24.9)	23.7 (22.3, 25.2)	0.034
Socioeconomic status and lifestyle data				
High educational attainment (*n*, %)	69	23 (67.6%)	24 (68.6%)	>0.9
Duration of veganism (years)	69	4.9 (3.1, 11.1)	0	
Physical activity (*n*, %) ^a^	69			0.7
Inactive		5 (14.7%)	4 (11.4%)	
Active		29 (85.3%)	31 (88.6%)	
Smoking status (*n*, %)	69			0.2
Non-smoker		23 (67.6%)	21 (60.0%)	
Ex-smoker		8 (23.5%)	5 (14.3%)	
Current smoker		3 (8.8%)	9 (25.7%)	
Menopausal status (*n*, %) ^b^	33			0.5
Pre/peri		13 (76.5%)	14 (82.4%)	
Post		3 (17.6%)	3 (17.6%)	
Dietary intake data				
Total energy (MJ/d)	69	9.6 (7.7, 11.7)	10.0 (8.9, 11.5)	0.4
Protein (%en)	69	12.2 (10.9, 15.7)	14.6 (12.3, 16.6)	0.019
Protein (g/kg body weight/d)	69	1.0 (0.9, 1.4)	1.2 (1.1, 1.5)	0.06
Alcohol (g/d)	69	0.05 (0.00, 2.00)	1.16 (0.02, 13.7)	0.026
24 h urinary excretion data				
uPRAL (mEq/24 h)	69	−37.3 ± 31.5	−2.8 ± 22.5	<0.0001
Urine volume (L/24 h)	69	2.2 (1.6, 2.9)	1.9 (1.5, 2.6)	0.3
Chloride (mmol/24 h)	69	132 (94, 190)	147 (102, 203)	0.6
Sulfate (mmol/24 h)	69	14.7 (10.1, 18.9)	20.8 (13.6, 24.2)	0.012
Phosphate (mmol/24 h)	69	18.9 (14.1, 24.0)	26.5 (21.1, 39.0)	0.0004
Sodium (mmol/24 h)	69	143 (102, 184)	138 (100, 198)	0.9
Potassium (mmol/24 h)	69	82 (59, 106)	69 (61, 107)	0.7
Calcium (mmol/24 h)	69	2.9 (1.8, 4.5)	4.1 (3.1, 5.8)	0.046
Magnesium (mmol/24 h)	69	5.4 (4.0, 7.1)	4.2 (3.3, 6.8)	0.1
Urinary pH	69	6.7 (6.4, 7.1)	6.2 (5.9, 6.4)	<0.0001
Bone health parameters ^c^				
BUA (dB/MHz)	68	112 ± 11	118 ± 11	0.037
SOS (m/s)	68	1584 ± 27	1593 ± 40	0.3
SI	68	98 ± 13	104 ± 17	0.1

Results presented as mean ± SD, median (25th, 75th percentile) or absolute (relative) frequencies. BUA—broadband ultrasound attenuation; SOS—speed of sound; SI—stiffness index. ^a^ Moderate to high physical activity equals > 33.75 MET h/week of walking, cycling and sports. ^b^ *n* = 33 due to 1 missing menopausal status among the total of 34 female participants; % calculated based on the total *n* of females among vegans and omnivores, respectively; postmenopausal status includes operative menopause. ^c^ Reduced sample sizes (*n* = 68) result from exclusion of 1 participant due to a missing QUS measurement.

**Table 2:** Association of uPRAL with quantitative ultrasound parameters of bone health (*n* = 68) presented as linear trends and least-squared means of bone health parameters in uPRAL tertiles.

	Predictor: uPRAL					
	T1 (*n* = 22) −53.6 (−67.0, −44.1) ^a^	T2 (*n* = 24) −15.2 (−21.5, −10.9) ^a^	T3 (*n* = 22) 14.1 (4.0, 20.2) ^a^	*β*_trend_	*SE*_trend_	*p*_trend_
Vegans/Omnivores ^b^	19/3	10/14	5/17			
BUA (dB/MHz)						
Model A	114 (109–119)	113 (109–117)	118 (113–123)	+0.024	0.042	0.6
Model B	116 (111–121)	112 (108–117)	116 (111–121)	−0.031	0.048	0.5
Model C	116 (111–121)	113 (109–117)	116 (111–121)	−0.041	0.046	0.4
Model D	116 (111–121)	114 (109–118)	115 (111–120)	−0.030	0.048	0.5
SOS (m/s)						
Model A	1584 (1570–1599)	1584 (1570–1598)	1597 (1583–1611)	+0.166	0.128	0.2
Model B	1586 (1570–1603)	1584 (1570–1598)	1595 (1580–1611)	+0.129	0.152	0.4
Model C	1587 (1571–1602)	1585 (1572–1599)	1593 (1578–1608)	+0.100	0.147	0.5
Model D	1587 (1571–1604)	1586 (1572–1600)	1592 (1576–1607)	+0.127	0.159	0.4
SI						
Model A	100 (93–106)	99 (92–105)	106 (99–112)	+0.062	0.058	0.3
Model B	102 (94–109)	98 (92–104)	104 (97–111)	+0.015	0.068	0.8
Model C	102 (95–109)	99 (93–105)	103 (96–110)	+0.001	0.065	>0.9
Model D	102 (95–109)	100 (94–106)	102 (96–109)	+0.015	0.069	0.8

Presented are least-squared means (95%-CI) of bone health outcomes in sex-specific tertiles of the predictor uPRAL unless otherwise indicated. Linear trends (*β*_trend_, *SE*_trend_, *p*_trend_) were obtained in general linear models with uPRAL as a continuous variable. BUA—broadband ultrasound attenuation; SOS—speed of sound; SI—stiffness index; T—tertile. Model A: unadjusted; Model B: adjusted for veganism (yes/no); Model C: additionally adjusted for sex, age (y); Model D: additionally adjusted for BMI (kg/m^2^), smoking status (current vs. ex vs. non-smoker), moderate to high physical activity (yes/no), alcohol intake (g/d), protein intake (g/d), 24 h urinary calcium excretion (mmol/24 h). ^a^ Medians (25th, 75th percentiles) for predictor uPRAL in tertiles. ^b^ Numbers of vegans and omnivores in tertiles of predictor uPRAL.

## Data Availability

The datasets generated and/or analyzed during the current Risks and Benefits of a Vegan Diet study are not publicly available due to provisions of the data protection regulations.
